# Next Decade’s AI-Based Drug Development Features Tight Integration of Data and Computation

**DOI:** 10.34133/2022/9816939

**Published:** 2022-01-17

**Authors:** Yunan Luo, Jian Peng, Jianzhu Ma

**Affiliations:** ^1^Department of Computer Science, University of Illinois at Urbana-Champaign, Urbana, IL 61801, USA; ^2^School of Computational Science and Engineering, Georgia Institute of Technology, Atlanta, GA 30332, USA; ^3^Institute for Artificial Intelligence, Peking University, Beijing 100871, China

Traditional drug development heavily relies on human-derived rational and effort to detect the functional mechanisms of diseases, identify druggable targets, and design lead compounds to hit the targets. Despite our progress in understanding human diseases and the advances in biotechnology, the search for novel therapeutics remains a time-consuming and costly process. With the recent tremendous success of artificial intelligence (AI) in various domains, AI-based drug development is poised to become a revolutionary force in the pharmaceutical sector and is expected to fundamentally change the traditional trial-and-error design process (Figure 1(a)). 

**Figure 1:**
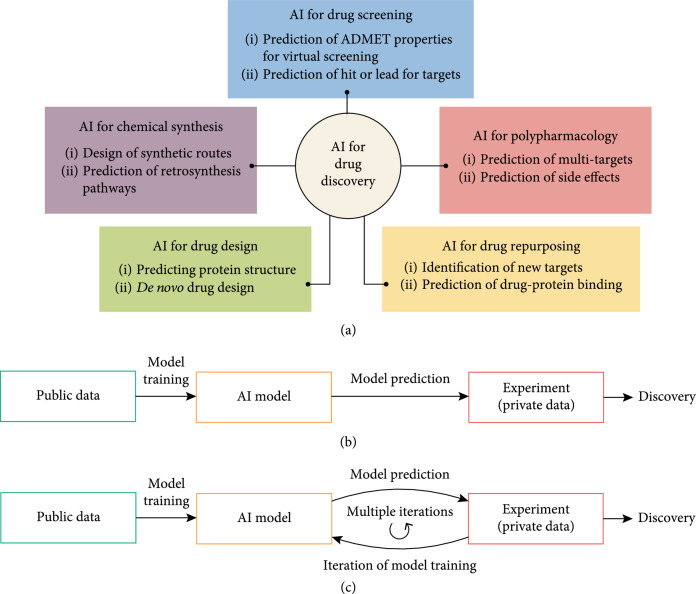
AI for drug development. (a) AI can be used for drug development in different ways, including drug screening, polypharmacology, drug repurposing, chemical synthesis, and drug design [6]; ADMET: absorption, distribution, metabolism, elimination, toxicity. (b) Illustration of the traditional paradigm of AI-based drug development where AI and data generation are connected in a linear way. (c) Illustration of an active learning paradigm of AI-based drug development where AI and data generation form an iterative feedback loop.

Promising progress has been made in using AI for drug design. For instance, Insilico Medicine has applied deep learning techniques to discover potent inhibitors of discoidin domain receptor 1 (DDR1) [1]. UK’s Excientia has developed the world’s first AI-designed drug to enter phase 1 clinical trials in 2020, along with another two clinical-trial drugs in 2021. DeepMind’s AlphaFold [2] is yet another revolutionary breakthrough. Its unprecedented structure prediction accuracy can make a potential impact on structure-based drug design, especially for new targets that have not been solved structurally [3]. 

Despite the above exciting results in AI-based drug development, we are still far from certain that these early results could be translated to more effective drugs with a high success rate. The critical problem in drug development is the failure of the candidate molecules in clinical trials. Increasing the success rate in clinical trials is arguably the most profound factor in reducing the overall cost and outweighs the saving in other stages. The main challenge is to identify candidate molecules that are not only effective but also do not cause toxicity and other unexpected side effects. How can AI help with this? We need to rethink how AI should be integrated into the drug development pathway. In this perspective, we highlight two paradigms, active learning and interpretable AI, as promising future directions for AI-based drug development.

As a data-driven approach, the advantage of AI-based drug development is the capability of mining large-scale data and extracting patterns that might be less salient or too complex to humans. Therefore, how to really harness the value of data is the key to building successful AI models. A conventional and popular paradigm leveraging AI for the drug development process is to linearly invoke AI models from experimental data (e.g., data from high-throughput screening, assay/animal validations) for the purpose of prediction (Figure 1(b)). In this paradigm, AI models are typically used to screen virtual libraries of potential molecules and predict those that might have the desirable properties, which could be validated by downstream experiments. The major limitation of this linear paradigm is the efficiency of new discovery: the model’s predictions, although potentially informative, are only “educated guesses” until experimentally validated [4]. Unfortunately, it is often infeasible to thoroughly validate the predictive models with the tremendous effort of high-throughput screenings. To address this challenge, a promising solution that has gradually gained recognition is active learning, a subfield of AI that tightly integrates data and computation to improve predictive models. Active learning transforms the traditional AI-based development from a linear process to an iterative paradigm (Figure 1(c)). Rather than using AI and experimental biology as isolated tools in the process, active learning creates an interactive feedback loop between the two that informs each other to improve the overall outcome. For example, after training on the initial public dataset and predicting the property of molecules in a virtual library, the AI model might plan the next steps by proposing a handful of molecules, including those expected to succeed as well as those predicted to fail, for experimental validation. What makes active learning appealing is the iterative cycle where drug developers can iteratively leverage the AI-generated hypotheses to design and execute the next round of experiments: AI models can first suggest molecules to synthesize and validate, the validation results are then used to further correct or reinforce the model’s prediction ability, and the model’s new predictions inform another cycle of testing and analysis. These data-computation interactions thereby more efficiently guide drug developers to discover novel molecules with desirable properties. Working as a combination of a hypothesis-generator and a validation engine, active learning can eliminate “bad” candidates more quickly than the linear paradigm and better focus experts’ creativity and effort on candidates that are more likely to succeed. Furthermore, the data-computation loop also allows generating data that are specifically tailored to AI applications. In contrast, existing data have limitations related to quantity or quality and may not be suitable for every AI algorithm. Several AI-powered drug discovery companies such as Insitro have been applying this paradigm for integrating AI and data generation, not prioritizing one over the other, to discover new therapeutics [5]. 

In addition to the capability to fully exploit the value of data, another advantage of this paradigm is the synergy between AI and human intelligence, where medicinal chemists can guide AI to be more accurate and creative and AI can augment the experts’ capabilities to discover improved and novel medicine. However, this requires AI models that are interpretable to humans, i.e., revealing the internal rationale behind a prediction. As AI-supported drug design is a high-stack decision-making problem, explanations of why the model makes a certain prediction are highly demanded, even though the model’s prediction accuracy is impressive. Blending the mechanistically interpretable and high-accuracy models is considered critical to accelerated drug discovery with AI [7, 8]. Knowing the mechanistic explanation (interpretation) of successful AI-designed molecules would reveal insights that can be potentially generalized for future drug designs. Designing new drugs essentially is a problem of optimizing pharmacological activities by varying molecular structures, and it is important to identify structural elements that are relevant (determinants). For example, in AI-based antibody design, a model that uncovers interactions existing between the antibody and antigen residues would explain the structural basis of high-performance antibodies. Most of the modern AI models, such as deep neural networks, are “black boxes,” eluding accessibility by the human mind, which might prevent scientists from assessing the novelty or reliability of the AI-generated hypothesis. Take Insilico’s AI-discovered DDR1 inhibitor as an example: it was found that this compound is highly similar to the marketed drug ponatinib [9]. Ponatinib is a DDR1 inhibitor that targets many other kinases and was assigned with a boxed warning by US FDA because of its potential side effects. Given its striking similarity to ponatinib, the selectivity and safety of Insilicon’s compound should be questioned. This example highlights the importance of the interpretability and transparency of AI models for drug discovery [7]. Preferably, the AI model should unveil how it reaches a particular prediction, e.g., based on which training molecules. Knowing the insight and logic of AI’s prediction will help scientists avoid correct predictions for wrong reasons and reveal the caveats that are too subtle to the human mind [7]. Explainable AI is an active direction in the machine learning community, and its applications to drug development will be beneficial for creating the iterative cycle of AI, experimental biology, and human feedback. 

Drug development, for decades, has been time-consuming and expensive. The impressive advances of AI shifted our mindset for a new paradigm to design drugs [8]. We expect that the next decade of AI-based drug development will feature a deep engagement of interpretable AI approaches and active learning algorithms, which iteratively improve the workflow and generate interpretable insights that scientists can monitor, analyze, and understand for every stage in drug development. 

## Data Availability

No data were used to support this study.
